# Use of alarm features in predicting significant endoscopic findings in Nigerian patients with dyspepsia

**DOI:** 10.11604/pamj.2019.34.66.18848

**Published:** 2019-10-02

**Authors:** Emuobor Aghoghor Odeghe, Oluwafunmilayo Funke Adeniyi, Ganiyat Kikelomo Oyeleke, Samuel Olalekan Keshinro

**Affiliations:** 1 Department of Medicine, College of Medicine, Lagos University Teaching Hospital, Lagos, Nigeria; 2 Department of Paediatrics, College of Medicine, Lagos University Teaching Hospital, Lagos, Nigeria; 3 Arrive Alive Diagnostic and Imaging Services, Surulere, Lagos, Nigeria

**Keywords:** Dyspepsia, alarm features, endoscopy, Nigeria

## Abstract

**Introduction:**

Dyspepsia is prevalent in the community. Guidelines recommend early endoscopy in dyspeptic patients who are older than 55 years, or have alarm features. There is a lack of data on endoscopy in patients with alarm features in Nigeria.

**Methods:**

A retrospective study of the endoscopic findings in adults with dyspepsia and alarm features, between August 1^st^ 2017 and July 31^st^ 2018 in Lagos, Nigeria. Data were analysed using Statistical Package for Social Sciences, version 23.0. The sensitivity, specificity, positive predictive value, and negative predictive value of the alarm features were calculated.

**Results:**

One hundred and fifty-nine gastroscopies were performed during this period, mean age was 47.8 (±14.4) years, 49.1% were male. Dyspepsia was the commonest indication for endoscopy (80.5%), 60.2% of the dyspeptics had at least one alarm feature. The most frequent dyspeptic symptom was epigastric pain/burning sensation (75%), while the commonest alarm features were recent onset dyspepsia in a patient over 45 years (79%) and unexplained weight loss (28.6%). Endoscopy was normal in 26%. The most frequent significant endoscopic findings were gastritis (49%) and gastric ulcer (17%) and they were not associated with alarm features. Upper gastrointestinal bleeding, persistent vomiting and odynophagia were specific for significant endoscopic findings. The pooled sensitivity, specificity, positive predictive value, and negative predictive value of the alarm features were 65%, 49%, 71% and 41% respectively.

**Conclusion:**

Patients with dyspepsia and upper gastrointestinal bleeding, persistent vomiting or odynophagia, should be referred for prompt upper GI endoscopy.

## Introduction

Dyspepsia is defined by the Rome criteria as chronic or recurrent pain or discomfort that is located in the upper abdomen, and includes symptoms such as early satiety, bloating, upper abdominal fullness or nausea [1]. Dyspeptic symptoms constitute a significant proportion of outpatient gastrointestinal (GIT) consultations. In a study of GIT outpatient consultations at the Lagos University Teaching Hospital (LUTH), patients with dyspepsia comprised 31.6% of the 1663 patients seen [2]. In a study in a rural community in north eastern Nigeria, a prevalence of 26% was documented [3]. Various guidelines recommend that dyspeptic patients over 55 years, and those with alarm features (bleeding, anaemia, early satiety, unexplained weight loss, progressive dysphagia, odynophagia, persistent vomiting, family history of GIT cancer, previous oesophago-gastric malignancy, previous documented peptic ulcer, and lymphadenopathy) undergo prompt endoscopy to rule out peptic ulcer disease, oesophagogastric malignancy and other rare upper GIT disease [4, 5]. Several studies have sought to determine the utility of alarm features in predicting serious upper GIT disease, with conflicting results [4-6]. In a systematic review and meta-analysis of 15 studies with 57,363 patients, the overall sensitivity and specificity of the alarm features in predicting GI malignancy on endoscopy were 0-83% and 40-98% respectively, while the sensitivity and specificity with just clinical diagnosis of upper GI malignancy by a physician were 11-53% and 97-98% respectively, leading the authors to conclude that the alarm features only have limited value for predicting underlying malignancy [6]. Other workers have documented a similar finding [7]. In contrast, some authors document a good predictive value of the alarm features in patients with dyspepsia when endoscopy is performed, and support the use of endoscopy in these patients [8-10]. Reports from Asia support endoscopic evaluation of dyspeptic patients older than 35 years even in the absence of alarm features [11]. Endoscopic findings that have been reported in adults over 45 years with these two features include erosive oesophagitis, Barrett's oesophagus, gastric or duodenal ulcer disease, and gastro-oesophageal malignancy [12]. In Nigeria, there are only a few reports addressing this subject. Olokoba *et al.* reported three patients, less than 40 years, with dyspepsia and alarm features, who were found on endoscopy to have antral masses, which were reported as gastric adenocarcinoma on histology [13]. The aim of this study was to describe the pattern of alarm features seen in adults with dyspepsia referred for endoscopy at two private facilities in Lagos and to document the endoscopic findings in these patients. The predictive value of alarm features in dyspeptic patients undergoing endoscopy was also documented.

## Methods

This was a retrospective study of the endoscopic records of all adult patients who had gastroscopy for dyspepsia and alarm features between August 1^st^ 2017 and July 31^st^ 2018 at the Endoscopy suites of two private endoscopy centres in Lagos. The two private endoscopy centres, one of which is located in the mainland area, and the other in the island area of Lagos state, receive patients for endoscopy procedures from within the state and beyond, and offer diagnostic and therapeutic GI endoscopy services to adults and children. An alarm feature was defined as any of the following: recent onset dyspepsia in a patient over 45 years, gastrointestinal bleeding, early satiety, unexplained weight loss, progressive dysphagia, odynophagia, persistent vomiting, personal or family history of gastrointestinal malignancy and previous documented peptic ulcer. The inclusion criterion was adult patients (>18 years) who were referred for endoscopic evaluation of dyspepsia. Patients who were younger than 18 years were excluded. Ethical approval was obtained from the Lagos University Teaching Hospital Health Research Ethics Committee before commencement of the study. The following data were retrieved and entered into a proforma created for this study: basic demographics, the indication for the procedure, dyspeptic symptoms present, alarm features present, and the endoscopic findings. The endoscopic procedures were performed after an overnight night by three Gastrointestinal Endoscopists using Olympus GIF160 video endoscopes, and endoscopic diagnoses were based on visual examination. Subjects with significant endoscopic findings were referred to the Gastroenterology clinic of LUTH for further evaluation. Data were analysed using SPSS version 23 (SPSS Statistics for Windows, version 23.0, IBM Corp and USA). Basic descriptive statistics were performed and displayed as frequency tables. Categorical data were compared using χ2 test or Fisher exact test, where appropriate. P-values <0.05 were considered significant.

## Results

General characteristics of the study participants: Table 1 summarises the characteristics of the study population. One hundred and fifty-nine gastroscopic procedures were performed during the period under review. The mean age of the study participants was 47.8 (±14.4) years, with a range of 19-80 years, and peak age of 30-39 years. The mean body mass index (BMI) was 27.0 (±4.7) kg/m^2^, and more than a third (36.5%) of them were overweight or obese. Dyspepsia was present in 80.5%, and 60.2% of the dyspeptics had at least one alarm feature. The dyspeptic symptoms, and the alarm features present in the participants with dyspepsia are presented in Table 2. The presence of dyspepsia was significantly associated with the female gender (91% versus 69%, p=0.001). The most frequent dyspeptic symptoms were epigastric pain or burning (75%), heartburn (11.7%), regurgitation (11.7%), and feeling of a lump in the throat (9.4%). Less frequent were bloating (6.3%), belching (5.5%), postprandial fullness (3.9%), retrosternal discomfort (3.9%), and early satiety (0.8%). The common alarm features were recent onset dyspepsia in a patient over 45 years, unexplained weight loss and dysphagia. Many patients presented with multiple dyspeptic symptoms and alarm features. The most frequent significant endoscopic findings were gastritis and gastric ulcer; there was no association between the alarm features and any significant endoscopic finding (p=0.3), as shown in Table 3. The pooled sensitivity and specificity of any alarm feature for any significant endoscopic finding were 64.7% and 48.8%, the PPV and NPV were 71.4% and 41.2% respectively; while for the individual alarm features these were 0-65%, 49-100%, 0-100% and 33-41% respectively; these are shown in Table 4.

**Table 1:** General characteristics of the study participants

Characteristic	Frequency	%
Mean age (SD) years	47.8 (14.4)	
Gender Male	78	49.1
Female	81	50.9
Presence of dyspepsia	128	80.5
Dyspepsia with alarm features	77	60.2

**Table 2:** Distribution of dyspeptic symptoms and alarm features

Dyspeptic symptom, n=128 (80.5%)	
Epigastric pain	96 (75)
Heartburn, regurgitation	15 each (11.7)
Feeling of lump in the throat	12 (9.4)
Bloating	8 (6.3)
Belching	6 (4.7)
Postprandial fullness and retrosternal pain	5 each (3.9)
Early satiety	1 (0.8)
**Alarm features, n=77 (60.2%)**	
Recent onset dyspepsia in >45 years	61 (79.2)
Unexplained weight loss	22 (28.6)
Dysphagia	11 (14.3)
Upper gastrointestinal bleeding	9 (11.7)
Odynophagia	8 (10.4)
Lower gastrointestinal bleeding, persistent vomiting, positive faecal occult blood test, previous documented peptic ulcer disease	1 each (1.3)

N: number of patients (%)

**Table 3:** Association of alarm features with endoscopic findings

Endoscopy finding	Alarm feature (+) n (%) N=77 (60.2)	Alarm feature (-) n (%) N= 51 (39.8)	P value
Significant endoscopic finding	55 (71.4)	32 (62.7)	0.30
Normal endoscopy	22 (28.6)	19 (37.3)	

Alarm feature: recent onset dyspepsia in a person > 45 years, odynophagia, progressive dysphagia, upper gastrointestinal bleeding, recurrent vomiting, unexplained weight loss.

Significant endoscopic finding: oesophagitis, gastritis, duodenitis, peptic ulcer, upper gastrointestinal tract polyp/cancer

**Table 4:** Sensitivity, specificity, PPV, NPV of the alarm features for significant endoscopic finding

Alarm features	Sensitivity	Specificity	PPV	NPV
Dysphagia	8.2	90.7	63.7	33.3
Haematochesia	0	97.7	0	33.1
Odynophagia	7.1	95.3	75	34.2
Persistent vomiting	0	97.7	0	33.1
Positive faecal occult blood test	1.2	100	100	33.9
Previous documented peptic ulcer	1.2	100	100	33.9
Recent onset in a patient over 45 years	51.8	60.5	72.1	38.8
Unexplained weight loss	18.8	86	72.7	34.9
Upper gastrointestinal bleeding	8.2	95.3	77.8	34.5
Pooled values	64.7	48.8	71.4	41.2

PPV: Positive Predictive Value, NPV: Negative Predictive Value

## Discussion

In this study, majority of the study participants were over 40 years of age, overweight, with an equal number of males and females. This is similar to the findings of other studies [14-18]. Some authors included paediatric data in their studies, and thus the mean age obtained was lower than what was obtained in this study [19-21]. Overweight is increasingly becoming a problem in the urban areas of many developing countries as is also observed in other studies [17, 22, 23], and this may be due to the adoption of a western lifestyle. The common dyspeptic symptoms were epigastric pain/burning, heartburn, regurgitation, and feeling of a lump in the throat. This finding was also observed in a study from the United States [24]. Less common symptoms were bloating, belching, postprandial fullness and early satiety. There were more females than males with dyspepsia (p = 0.001). Many studies have documented that dyspepsia is very common in the community [3, 25], and it remains the commonest reason for Gastroenterology consultations [2, 26, 27]. It is also the commonest indication for endoscopy [14, 21, 28-34], and a similar observation was made in the present study. Sixty percent of the dyspeptic patients had at least one alarm feature. This is similar to findings by other investigators [24, 30, 35], however, lower figures have been reported by some studies [10, 19, 21, 36, 37]. The high prevalence of alarm features in our study may be attributed to late presentation and poor health seeking behaviour. This may be due to a lack of affordable health care in the country, with many patients resorting to the use of traditional or herbal mixtures, and over-the-counter drugs (OTC) for their health complaints. Thus, many patients may delay seeking medical care until complications occur, and these may manifest as any of the alarm features. This observation may be supported by the age of presentation of the study participants (majority were above 40 years), and the fact that the commonest alarm feature was recent onset dyspepsia in a person over 45 years. Recent onset dyspepsia in adults over 55 years was documented as the commonest alarm feature in another study [15], while other workers have reported that anaemia, vomiting and gastrointestinal bleeding were the most frequent alarm features in their study cohorts [19, 24, 36]. These differences in alarm features may be due to different patient characteristics.

Other alarm features seen in this study were unexplained weight loss, dysphagia, and upper gastrointestinal bleeding. This is similar to what has been reported in other studies [19, 24, 36]. Non-steroidal anti-inflammatory drugs (NSAIDs) are commonly misused in our society for inappropriate symptoms such as tiredness and inability to sleep, and this may account for the high frequency of upper gastrointestinal bleeding in our study. In terms of endoscopic findings, 28.6% of the patients with dyspepsia and alarm features had normal findings on endoscopy, giving an endoscopic diagnostic yield of 71.4%. This is similar to reports from India (35%), Ghana (32%), and China (35%) [21, 28, 36], but in contrast to a study from the United Kingdom where the endoscopy was normal in 73% [15]. This difference may be because the investigators included the findings of gastritis and duodenitis as normal [15], in contrast to our study where they were classified as abnormal. The most frequent significant endoscopic finding in our patients with dyspepsia and alarm features was gastritis. This finding may be due to the high prevalence of Helicobacter pylori in our environment, and thus all participants were referred for H. pylori testing after the procedure. Other workers have reported a similar finding [24], while others report oesophagitis and peptic ulcer disease as the most frequent endoscopic finding [15, 21, 28]. Other significant endoscopic findings in this study were peptic ulcer disease, oesophagitis, gastroduodenal polyps, gastric cancer, duodenitis, and oesophageal cancer, as have been reported elsewhere [15, 24]. In this study, alarm features in the patients with dyspepsia were not associated with any significant endoscopic finding. This is in contrast to studies that report an association between alarm features and abnormal endoscopic findings, such as polyps, and cancers, [24, 28, 35], with gastric cancer being eight times more common in those with alarm features than in those without [28]. Other studies have reported that alarm features such as gastrointestinal bleeding, anaemia, dysphagia, weight loss and increasing age were positive predictors of oesophago-gastric malignancy [15, 18, 36, 38, 39]. These differences may be due to different patient characteristics, as well as the local prevalence of gastric cancer. The pooled sensitivity and specificity of the alarm features for any significant endoscopic finding were moderate. This is similar to findings from other studies where alarm features were found to be of low sensitivity in predicting upper gastrointestinal malignancy [7, 10]. Upper gastrointestinal bleeding, persistent vomiting and odynophagia however had >95% specificity for significant endoscopic findings (oesophago-gastric mass, oesophagitis and peptic ulcer disease). One study found a high specificity of dysphagia in predicting upper gastrointestinal malignancy [10], while another reported that bleeding, weight loss, dysphagia and age over 45 years predicted 97% of cancers [40]. Limitations of this study include its retrospective design, lack of testing for H. pylori endoscopically, and inability to confirm the diagnosis of cancer histologically because the investigators had no access to the histology reports of the study participants who returned to their primary physicians after the endoscopy procedure.

## Conclusion

In this study, the common alarm features in patients with dyspepsia were recent onset dyspepsia in an adult older than 45 years, unexplained weight loss, dysphagia and upper gastrointestinal bleeding. Gastritis was the commonest endoscopic finding. The pooled sensitivity of the alarm features for any abnormal endoscopic finding was moderate at 65%, however, upper gastrointestinal bleeding, odynophagia and persistent vomiting were specific for oesophagitis, cancer and peptic ulcer disease. Therefore, dyspeptic patients with upper gastrointestinal bleeding, odynophagia or persistent vomiting should be referred for prompt endoscopy.

### What is known about this topic

- Dyspepsia is the most common indication for upper endoscopy;
- Increasing age, and alarm features may predict the presence of significant upper gastrointestinal disease.

### What this study adds

- The commonest alarm symptom in patients with dyspepsia in Nigeria is recent onset dyspepsia in a patient over 45 years;
- In Nigerian patients with dyspepsia, upper gastrointestinal bleeding, odynophagia and persistent vomiting are specific for the presence of significant upper gastrointestinal disease such as oesophagitis, peptic ulcer disease and malignancy, and these patients should be referred for prompt endoscopy.

## Competing interests

The authors declare no competing interests.
